# Google Trends on Human Papillomavirus Vaccine Searches in the United States From 2010 to 2021: Infodemiology Study

**DOI:** 10.2196/37656

**Published:** 2022-08-29

**Authors:** Akshaya Srikanth Bhagavathula, Philip M Massey

**Affiliations:** 1 Center for Public Health and Technology Department of Health, Human Performance, and Recreation, College of Education and Health Professions University of Arkansas Fayetteville, AR United States

**Keywords:** Google Trends, HPV vaccine, Google search, attitude, infodemiology, searches, United States of America

## Abstract

**Background:**

The human papillomavirus (HPV) vaccine is recommended for adolescents and young adults to prevent HPV-related cancers and genital warts. However, HPV vaccine uptake among the target age groups is suboptimal.

**Objective:**

The aim of this infodemiology study was to examine public online searches in the United States related to the HPV vaccine from January 2010 to December 2021.

**Methods:**

Google Trends (GT) was used to explore online searches related to the HPV vaccine from January 1, 2010, to December 31, 2021. Online searches and queries on the HPV vaccine were investigated using relative search volumes (RSVs). Analysis of variance was performed to investigate quarterly differences in HPV vaccine searches in each year from 2010 to 2021. A joinpoint regression was used to identify statistically significant changes over time; the α level was set to .05.

**Results:**

The year-wise online search volume related to the HPV vaccine increased from 2010 to 2021, often following federal changes related to vaccine administration. Joinpoint regression analysis showed that HPV vaccine searches significantly increased on average by 8.6% (95% CI 5.9%-11.4%) across each year from 2010 to 2021. Moreover, HPV vaccine searches demonstrated a similar pattern across years, with search interest increasing through August nearly every year. At the state level, the highest 12-year mean RSV was observed in California (59.9, SD 14.3) and the lowest was observed in Wyoming (17.4, SD 8.5) during the period of 2010-2021.

**Conclusions:**

Online searches related to the HPV vaccine increased by an average of 8.6% across each year from 2010 to 2021, with noticeable spikes corresponding to key changes in vaccine recommendations. We identified patterns across years and differences at the state level in the online search interest related to the HPV vaccine. Public health organizations can use GT as a tool to characterize the public interest in and promote the HPV vaccine in the United States.

## Introduction

Human papillomavirus (HPV) is the most common sexually transmitted infection in the United States, and certain strains are associated with the majority of cancers of the cervix (90%), anus (90%), vagina and vulva (70%), penis (60%), and oropharynx (71%) [1]. In the United States, with nearly 80 million people currently infected with HPV and an estimated 14 million new cases each year, there is a significant burden of HPV-associated cancers [2]. Vaccination against HPV is highly effective at preventing HPV-related cancers, and the US Advisory Committee on Immunization Practices (ACIP) recommends two doses of the HPV vaccine for males and females aged 9-14 years, with catch-up doses recommended up to age 26 [3]. The US Department of Health and Human Services has set a goal to increase the proportion of adolescents who receive the recommended doses of the HPV vaccine to 80% by 2030 [4]. In 2020, up-to-date HPV vaccine coverage among adolescents remained below this mark at 58.6%; however, coverage was up from 54.2% in 2019 [5]. Although the HPV vaccine is safe, effective, and widely available, rates of HPV vaccine coverage in the United States remain suboptimal.

In the internet age, Google searches represent a common approach for discovering information online [6] and the HPV vaccine is one of the most widely discussed vaccinations on the internet [7]. Existing research on the HPV vaccine and social media using various platforms such as YouTube [7,8], Facebook [9], Instagram [10], and Twitter [11,12] have shown that a sizable proportion of HPV vaccine–related misinformation has created a negative perception of the HPV vaccine by the public [13]. During the first decade of HPV vaccine availability, research suggests that its representation on the internet is both positive and negative, with a growing number of false conspiracies and myths circulating [14].

Google Trends (GT) is a popular tool used to analyze online search behavior and search queries in the field of big data analytics in health care and public health research [15]. GT can show changes in online interest for any selected term in any country or region over a selected time period, and can also compare different regions simultaneously [16]. Data from GT have proven to be valuable to monitor health information–seeking behavior trends, often contributing to predictions or detection of outbreaks [17-21]. The emerging discipline of “infodemiology” focuses on these online behaviors, examining data from the internet, including GT, and is defined as “the science of distribution and determinants of information in an electronic medium, specifically the Internet, or in a population, with the ultimate aim to inform public health and public policy” [22].

To date, several studies have examined HPV vaccine–related misinformation [6-11], vaccine hesitancy [23], and arguments circulating on the internet [10,12]. However, there has been little to no research that has used the data of GT to look exclusively at online interest in the HPV vaccine based on search behavior. The purpose of this study was to characterize US public online searches and queries related to the HPV vaccine from 2010 to 2021, and determine the year-over-year changes in searches as well as differences across US states.

## Methods

### Data Collection

We collected monthly search volumes and search queries for the term “HPV vaccine” from GT between January 1, 2010, and December 31, 2021; the GT data retrieval period was from November 1, 2021, to January 31, 2022. GT provides a public database of the proportion of searches of a selected query performed on Google Search, and presents the data as a relative search volume (RSV) in a normalized format. The data can be delineated by specific topics and search terms, time and year, and location. Specific to each search term, the RSV value ranges from 0 (minimal to no interest) to 100 (high popularity) based on the term’s search volume. An RSV value of 100 indicates the maximum search interest for the time and location selected relative to that specific term.

GT enables exploring online searches at different time intervals and retrieval queries for any keywords entered in the Google search engine. Using this technique, we retrieved monthly online search queries and normalized RSVs related to the HPV vaccine across states in the United States. GT allows for queries of both “search terms” and “search topics.” The “search terms” query provides the results for all keywords that fall within the category and the “search topic” query renders the results of a group of terms that share the same concept in any language [16]. We used both search terms and search topics to query results for “HPV vaccine.”

We used the framework described by Mavragani and Ochoa [24] for the region selection and time period selection to retrieve query data from GT. Briefly, we searched for the keyword “HPV vaccine” at the country level (ie, the entire United States) to understand the overall RSVs in each year. Subsequently, using this information, we retrieved RSVs at the state level. All queries were searched between January 1, 2010, and December 31, 2021. The time periods demonstrating high-value RSVs were further investigated by checking with news bulletins or the scientific literature to identify any events associated with these same time periods.

### Statistical Analysis

We plotted a line chart to describe “HPV vaccine” search trends from January 1, 2010, to December 31, 2021. The annual mean (SD) is used to summarize the online searches for each year between 2010 and 2021. One-way analysis of variance followed by the Tukey posthoc test was performed to identify overall and quarterly differences in HPV vaccine searches in each year between 2010 and 2021. A joinpoint regression analysis was performed for each year to analyze the time trend in the GT data using the Joinpoint Regression program (version 4.9.1.0) developed by the National Cancer Institute [25]. This software analyzes trends by regression modeling while searching for temporal trend changes at time points called “joinpoints,” and estimates the regression function from previous joinpoints [26]. The number of joinpoints is obtained using a permutation test via Monte Carlo resampling [26] and the analysis criteria were set to find up to three joinpoints. The monthly percentage changes (MPCs) or annual percentage changes (APCs) between trend-change points were determined with their 95% CIs.

## Results

### Trends in RSVs Related to HPV Vaccine

**Figure 1:**
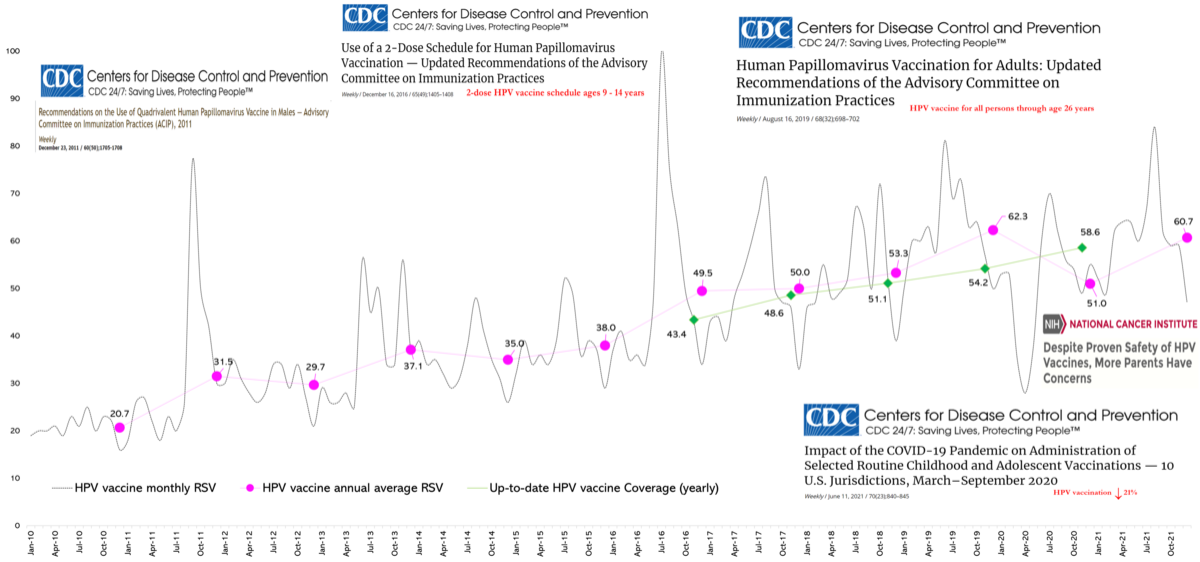
Human papillomavirus (HPV) vaccine–related relative search volumes (RSVs) on Google Trends from 2010 to 2021 in the United States with the corresponding timeline of Centers for Disease Control and Prevention (CDC) guidelines for HPV vaccine administration.

Figure 1 shows the trends in HPV vaccine online searches from 2010 to 2021, including both the monthly and annual mean RSVs, as well as the up-to-date HPV vaccine rates among 13-17 year-olds in the United States from 2016 to 2020. An increase in searches was observed in October 2011, when the ACIP recommended routine use of the quadrivalent HPV vaccine for boys aged 11-12 years [27]. Between January 2012 and June 2016, there were minimal increases in HPV vaccine searches. The RSV for HPV vaccine reached the highest peak value of 100% (ie, the most popular time the search term was used in our data set from 2010 and 2021) in late 2016, when the ACIP updated the HPV vaccination recommendation to use a 2-dose schedule for boys and girls who initiate the vaccination series at ages 9-14 years [28]. In June 2019, the ACIP recommended a catch-up HPV vaccination for all individuals aged up to 26 years, and the RSV on HPV vaccine reached 81% at this time [3]. Further, in 2020, there was a dramatic decrease in the RSV (28%) during the early COVID-19 pandemic and a comparable situation was observed in the latter half of 2021. The highest annual mean RSVs were recorded in 2018 (62.3%) and 2021 (60.7%), and the lowest annual mean RSVs were recorded in 2010 (20.7%).

### Quarterly HPV Searches From 2010 to 2021

Table 1 demonstrates the quarterly RSVs of HPV vaccine searches in each year from 2010 to 2021. Online search interest differed significantly across quarters in the years 2014, 2016, and 2017. In 2014, the search interest in the third quarter (July 1-September 30) was significantly higher than that in the second quarter (April 1-June 30) and fourth quarter (October 1-December 31). In 2016, search interest in the third quarter was significantly higher than that in the first, second, and fourth quarters. In 2017, third-quarter search interest was significantly higher than that in the first and fourth quarters.

**Table 1:** Quarterly differences in relative search volumes on Google Trends for the term “HPV vaccine” from 2010 to 2021 in the United States.

Year	Relative search volume point estimate, mean (SD)					*F*^a^ (*df*=3)		*P* value
	January 1-March 31 (group 1)	April 1-June 30 (group 2)	July 1-September 30 (group 3)	October 1-December 31 (group 4)				
2010	19.6 (0.5)	21.0 (2.0)	22.0 (2.6)	20.3 (3.7)	0.463		.72	
2011	23.6 (4.9)	21.0 (2.6)	41.0 (31.3)	40.6 (10.0)	1.242		.36	
2012	32.0 (2.6)	27.3 (1.1)	32.3 (2.1)	27.3 (6.5)	1.588		.27	
2013	27.0 (1.7)	36.3 (17.1	43.0 (8.1)	42.3 (11.9)	1.306		.34	
2014	36.0 (2.6)	30.6 (1.5)	42.3 (5.2)	30.6 (4.5)	6.593		.02^b, c^	
2015	35.0 (3.6)	36.3 (2.5)	45.6 (8.5)	35.0 (5.2)	2.659		.12	
2016	37.6 (3.0)	39.3 (7.5)	79.3 (18.8)	42.0 (7.5)	9.014		.005^b, c, d^	
2017	42.0 (2.6)	53.3 (5.5)	63.0 (11.8)	42.0 (7.8)	5.192		.03^c, d^	
2018	49.3 (4.9)	49.6 (2.0)	60.0 (8.8)	54.3 (16.6)	0.780		.54	
2019	56.6 (5.7)	67.3 (12.0)	68.3 (5.0)	57.0 (7.0)	1.914		.21	
2020	47.0 (10.4)	42.0 (16.3)	62.6 (7.0)	52.6 (3.2)	2.166		.17	
2021	54.3 (6.8)	62.6 (2.3)	71.0 (11.5)	55.0 (6.9)	3.146		.09	

^a^One-way analysis of variance followed by Tukey posthoc test for multiple comparisons.

^b^Significant (*P*<.05) difference between group 2 and group 3.

^c^Significant (*P*<.05) difference between group 2 and group 4.

^d^Significant (*P*<.05) difference between group 3 and group 1.

### State-Level HPV Vaccine Searches and Changes in HPV Vaccine Searches

Table 2 describes the average RSV of HPV vaccine searches at the state level for each year from 2010 to 2021 as well as the average across all 12 years. The highest 12-year mean RSVs were observed in California, New York, Texas, Florida, and Massachusetts, whereas Delaware, North Dakota, South Dakota, Vermont, and Wyoming recorded the lowest HPV vaccine searches.

**Table 2:** “HPV vaccine” relative search volume on Google Trends by US states for each year from 2010 to 2021.

State	2010	2011	2012	2013	2014	2015	2016	2017	2018	2019	2020	2021	Mean (SD)
Alabama	18.5	17.9	25.5	29.4	35.3	42	35.2	32	58.6	53.9	43.3	40.1	36.0 (12.6)
Alaska	11	16.9	18.2	28.9	22.2	19.2	36.7	23.8	34.9	23.9	26.8	28.8	24.3 (7.5)
Arizona	22.8	19.7	45	45.3	36.4	41.7	40.7	35	42.8	48.8	45.2	55.1	39.9 (10.2)
Arkansas	15.5	27.2	31.7	30.9	23	35	32.1	35.7	30.2	37.7	39.9	41.2	31.7 (7.3)
California	57.8	26.2	70	42.8	61.3	67.8	53.4	61.8	60.3	64.5	74	79.4	59.9 (14.3)
Colorado	24.7	22	36.4	27.3	48.2	48.9	40.6	53.8	51.2	56.3	51.9	53	42.9 (12.3)
Connecticut	25.6	23.1	36.2	32.7	31.7	50	40.6	47	57.7	54.3	40	45	40.3 (11.0)
Delaware	8.2	20.8	15.5	20.2	19.4	24.3	19.9	28.8	25	29.7	33.3	32.6	23.1 (7.3)
District of Columbia	34.2	18.5	41.5	23.9	34.5	56.6	44.1	42.2	26.1	31.7	24.9	31.5	34.1 (10.6)
Florida	57.6	26.5	60.1	49.3	67	57	47	49.1	38.5	60.1	63.4	53.9	52.5 (11.3)
Georgia	42.5	27.3	54.3	48.1	48	41.8	50.8	52.7	52	57	57.8	50.6	48.6 (8.3)
Hawaii	21.9	37.5	29.1	27.2	43	32.3	32.2	34.7	39.4	33.3	40.5	34.5	33.8 (5.9)
Idaho	9.7	18.1	25.1	23.6	37.1	26.2	32.6	24.8	35.8	36.8	31.9	28.8	27.5 (8.1)
Illinois	41	26.1	56	41.6	39.7	56.6	48.3	50.8	51.9	59.4	33.9	60.7	47.2 (10.8)
Indiana	36.1	29.8	37.7	52.8	39.2	42.6	41	43.6	43	52.5	58.5	50	43.9 (8.2)
Iowa	8.4	26.3	28.1	29	42.8	35	44	35.9	36.1	40.1	42	38.3	33.8 (9.9)
Kansas	27.1	18	33.6	44.7	18.3	30.7	32.7	29	49.2	41	44.7	38.3	33.9 (10.1)
Kentucky	32.2	29.3	39.5	35.8	37.3	42	26.1	39.8	33.9	48.1	47	38.4	37.5 (6.6)
Louisiana	28.5	18.7	40.3	38.8	41.4	36.3	38.8	33.8	57.7	50.2	47.5	53.8	40.5 (10.9)
Maine	8.6	30.1	27.2	22.2	12.4	25	34.6	24.6	34.9	32.5	15.1	33.1	25.0 (8.9)
Maryland	39.1	25	48.4	46.5	45.9	39.9	43	35.6	43	56.7	51.6	64.5	44.9 (10.1)
Massachusetts	43.8	27.3	54.1	46.5	56.7	52.4	45.8	50.1	58.3	64.7	51.4	65.4	51.4 (10.2)
Michigan	34.4	24.3	41.2	32.8	35.9	57.5	46.7	53.5	58.2	58	62.3	64.8	47.5 (13.4)
Minnesota	40.8	18.5	40.5	53	50.9	38	36.5	45.7	43.3	57.6	50.6	53.7	44.1 (10.5)
Mississippi	18.2	18.5	28.7	32.5	21.1	22.4	34.7	31.1	38.6	39.4	34.1	49.3	30.7 (9.5)
Missouri	40	25.3	49.4	37.9	48.2	52.7	34.4	31.7	47.6	50.9	38.1	47	41.9 (8.6)
Montana	11.2	17.7	24.3	20.4	20.6	20	26	25.2	24.5	34.7	32.8	30.6	24.0 (6.6)
Nebraska	21.3	18.3	32	39.3	33.2	26	20.3	35.4	41.4	44.5	42.1	43.7	33.1 (9.6)
Nevada	21.8	14.7	29.2	35.2	38.4	39.7	38.1	39	42.3	41.5	38	59.7	36.5 (11.2)
New Hampshire	19.2	15.8	31	29.4	25.4	30	28	28.5	35	40	28	31.5	28.5 (6.4)
New Jersey	43.3	29.7	39.5	44.2	39	58.3	47.1	48.5	46.6	51.8	44.9	59.7	46.1 (8.3)
New Mexico	24	20.7	21.6	29.9	26	27.1	35.1	41.1	40.5	30.7	20.1	33.8	29.2 (7.3)
New York	51.2	25.3	47.3	58.7	51.4	66.6	55.6	62.1	62.6	56.4	60.6	72.4	55.9 (11.9)
North Carolina	34.7	17.6	51.4	51.4	48.7	61.4	44.4	54.3	56.7	54	53.8	61.6	49.2 (12.3)
North Dakota	9.5	22.7	25.1	18.4	17	26.7	25.3	24.6	30.2	29.1	21.2	23.9	22.8 (5.7)
Ohio	43.5	24.4	52.4	33	41.2	38.6	40.7	44.2	58.4	57.6	54.6	55.5	45.3 (10.6)
Oklahoma	23.2	14.6	37.5	39.5	39.7	46.6	43	34.2	50.5	41.1	38.9	43	37.7 (9.9)
Oregon	21.6	31.7	38.5	31.2	48.4	38.2	33.5	54.9	29.7	50.8	43.6	40.5	38.6 (9.7)
Pennsylvania	50.1	35	53.4	24.9	43.2	52.9	46.1	55.2	51.1	69.3	61.4	69.8	51.0 (13.0)
Rhode Island	21	18	28.7	22	21	10.8	37.9	27.3	30.3	39.5	37.4	32	27.2 (8.9)
South Carolina	19.9	32.3	39.3	47	39.3	37.7	31.4	43.7	43.2	45.3	50.6	53	40.2 (9.2)
South Dakota	12	16.7	23.6	14.7	20.5	16.4	25.8	28.1	31.4	26.1	27.5	26	22.4 (6.2)
Tennessee	27.6	26.3	35.2	41.4	46.9	33.4	42.8	39.2	50.5	52.2	49.7	58	41.9 (10.0)
Texas	55	22.1	52.7	55.2	51.2	63.8	50	52.5	65	53.6	60.5	61.5	53.6 (11.1)
Utah	31.4	25.2	36.3	28.8	31	33	32.8	41	21	46.1	49.3	48.3	35.4 (9.1)
Vermont	12.6	15.6	15.1	23.3	22.1	25.3	21.3	27.8	24.9	16.6	16.7	33.8	21.3 (6.2)
Virginia	59.1	25.8	61.5	43.4	30	38.2	44.8	55.1	58.8	59.3	58.9	62.8	49.8 (12.9)
Washington	40	16.7	53.3	41.3	47.6	51.1	37.7	54.7	56	64	51.4	57.9	47.6 (12.5)
West Virginia	12.8	26.1	16.8	29.8	33.9	23.1	29	29.3	43.8	41.5	33.7	39.4	29.9 (9.4)
Wisconsin	31.4	28.7	44.7	47	20.8	45.1	42.4	39.8	55.3	44.3	44.2	62.2	42.2 (11.2)
Wyoming	9.5	7.9	3.5	17.3	14	22.2	13.5	13.9	25.4	31.5	26.7	23.5	17.4 (8.5)

### Trends in HPV Vaccine Searches 2010-2021

**Figure 2:**
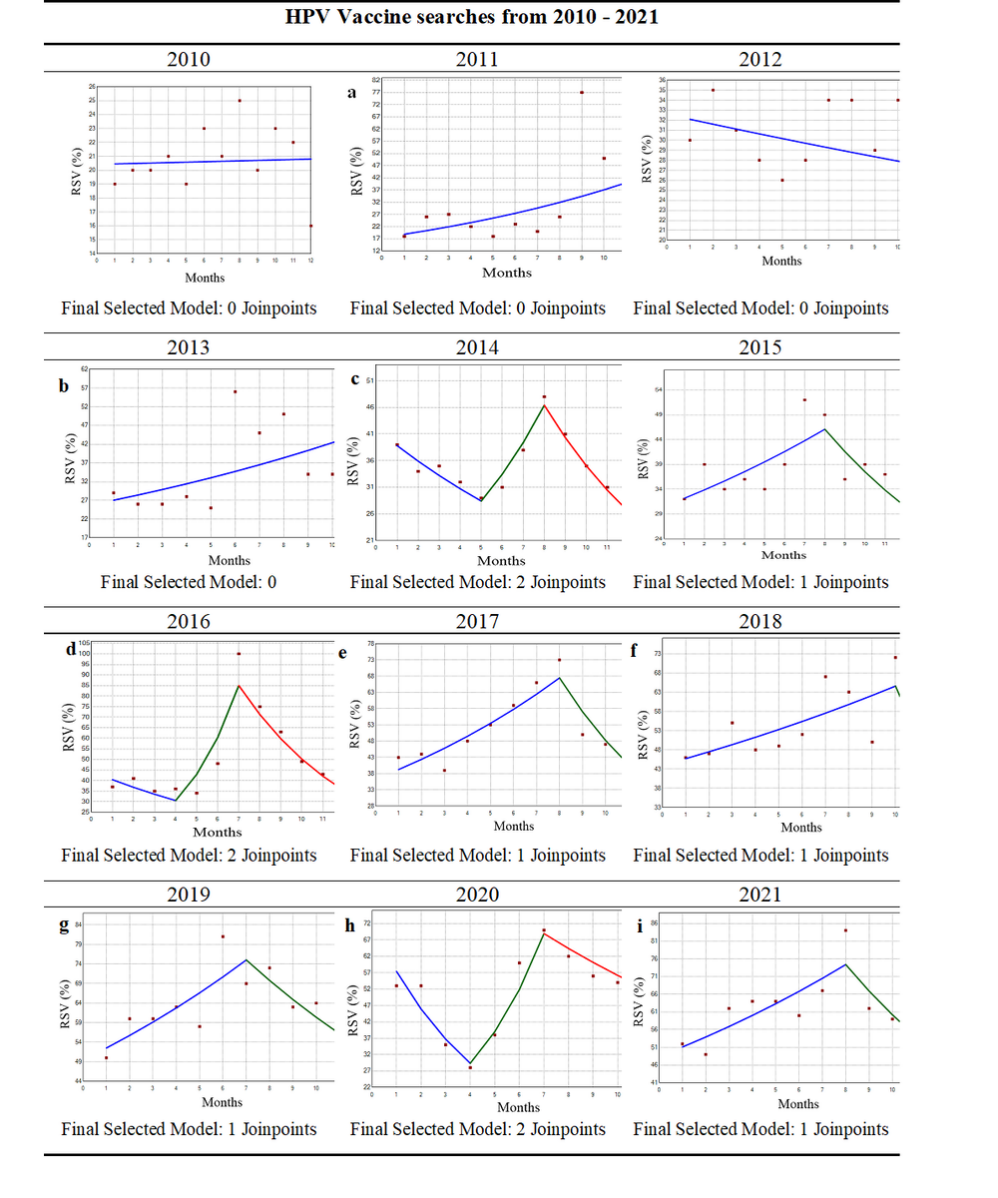
Joinpoint regression analysis indicating trends in "HPV vaccine" relative search volume (RSV) on Google Trends from 2010 to 2021 in the United States. Monthly percentage changes (MPCs) in the HPV vaccine RSVs are described in Table 2. The number of slopes is determined by the number of joinpoints identified by the analysis. Joinpoints are the time points when statistically significant changes in the linear slopes are noted.

The joinpoint regression plots are provided in Figure 2 and Table 3 gives the corresponding MPCs in the HPV vaccine searches in each full year from 2010 to 2021. Four out of the 12 years examined had no joinpoints (2010, 2011, 2012, 2013), suggesting no changes in search trends across the year. Five of the 12 years (2015, 2017, 2018, 2019, and 2021) had one joinpoint, suggesting two distinct time trends (one increasing and one decreasing) in searches during that year period. Three of the 12 years (2014, 2016, and 2020) had two joinpoints, suggesting three distinct trends, or changes, in searches. With respect to HPV vaccine searches, a common increasing trend across years in search volume (ie, search interest) was observed leading up to August.

Two joinpoints were noted in 2014, 2016, and 2020, all demonstrating similar patterns: a decrease in search interest early in the year, followed by an increase from April/May to August, and finishing with a decrease through December. Specifically, in 2014, there was a significant increase in the RSVs by 17.8% (*P*<.001) from May to August, followed by a significant decrease in the RSVs by 13.1% (*P*<.001) from August until December. The RSV search interest in 2016 demonstrated a very similar pattern. The beginning of 2020 demonstrated the largest significant downward trend of all time periods in the joinpoint regression, decreasing by 20% (*P*<.001) from January to April (ie, corresponding to the early COVID-19 pandemic time period). This sharp decrease was followed by an increase from April to July, although it was not significant.

To explore annual temporal changes in trends in HPV vaccine RSVs in the United States from 2010 to 2021, we estimated the APCs using joinpoint regression analysis and fit three models, allowing for no joinpoints, one joinpoint, and two joinpoints, respectively (Table 4). Model 1 showed that from 2010 to 2021, there was a significant annual average increase of 8.6% in RSVs. In Model 2, the joinpoint regression identified two trends: from 2010 to 2018 there was a significant annual average increase of 11.6% in RSVs, with an annual average decrease of –2.2% in RSVs from 2018 to 2021, although the decrease was not significant. In Model 3, the joinpoint regression analysis identified three separate trends, with only the period from 2012 to 2018 demonstrating a significant annual change in RSVs. Model 1 was the best-fitting model based on the permutation method [29].

**Table 3:** Monthly percentage changes (MPCs) in the “HPV vaccine” relative search volumes (RSVs) corresponding to the regression graphs (a–i) in Figure 2.

Regression graph in Figure 2	Year	Specified monthly period^a^	MPC in RSVs^b^
a	2011	1-12	7.80
b	2013	1-12	5.13
c	2014	1-5	7.50
c	2014	5-8	17.77
c	2014	8-12	–13.12
d	2016	4-7	40.73
d	2016	7-12	–16.14
e	2017	1-8	8.05
e	2017	8-12	–15.44
f	2018	1-10	3.91
f	2018	10-12	–21.73
g	2019	1-7	6.13
g	2019	7-12	–6.99
h	2020	1-4	–20.04
i	2021	1-8	5.51
i	2021	8-12	–10.05

^a^Nonsignificant monthly periods are not displayed.

^b^The MPC is significantly different from 0 at α=.05 in all periods.

**Table 4:** Joinpoint regression analysis showing changes in “HPV vaccine” relative search volume on Google Trends over time in the United States.

Segment	Period	Change year	Annual percentage change (95% CI)	*t* value	*P* value^b^
Model 1^a^	2010-2021	None	8.6 (5.9 to 11.4)	7.2	<.001
Model 2	2010-2018	2018	11.6 (7.3 to 16.1)	6.6	<.001
Model 2	2018-2021	2021	–2.2 (–18.4 to 17.2)	–0.3	.78
Model 3	2010-2012	2012	17.9 (–16.8 to 67.0)	1.3	.26
Model 3	2012-2018	2018	10.3 (2.0 to 19.3)	3.5	.03
Model 3	2018-2021	2021	–1.3 (–17.1 to 17.5)	–0.2	.85

^a^Final selected model, best fitting based on the permutation method.

^b^*P*<.05 indicates that the annual percentage change is significantly different from zero.

## Discussion

### Main Findings

To our knowledge, this is one of the first studies to examine US public online searches regarding the HPV vaccine using GT data. In analyzing the data on HPV vaccine–related online searches in the period from January 2010 to December 2021, we identified important trends, including an overall increase in online searches with noticeable spikes corresponding to key changes in vaccine recommendations. Overall, the joinpoint regression showed a significant average annual percentage increase of 8.6% in HPV vaccine search interest from 2012 to 2021, along with various time trends in HPV vaccine searches across years as well as within years. At the state level, the 12-year average annual HPV vaccine search interest in the United States was the greatest in California (59.9, SD 14.3), New York (55.9, SD 11.9), and Texas (53.6, SD 11.1), while Wyoming (17.4, SD 8.5) recorded the lowest interest in HPV vaccine searches.

### Comparison With Prior Studies

Although previous studies in the United States have explored the influence of social media on HPV vaccine communication [30-34], misinformation [10-14], social interactions, and HPV vaccination behavior [35-37], they also highlighted the need for specific strategies to counter misinformation spreading on the HPV vaccine. In our study, we documented an upward trend in HPV vaccine–related searches following federal changes related to vaccine administration. Despite the consistent evidence that the HPV vaccine is safe and effective, the up-to-date HPV vaccination coverage in 13-17–year-old adolescents was only 59% in 2020 [4]. Moreover, the percentage of parents who refused the HPV vaccine due to safety concerns nearly doubled [38]. Results from a recent study by Sonawane et al [39] showed that HPV vaccine safety concerns are increasing in 30 states. However, much of the information available on social media is not peer-reviewed or evidence-based, and researchers indicated that the information warning about the HPV vaccine is often comprised of innuendos, half-truths, or baseless propaganda [40]. Therefore, continuous monitoring of the trends specific to HPV vaccination across the national and regional landscape is essential to document online health information–seeking behaviors and potential safety concerns.

By using joinpoint regression, we found significant variation in the HPV vaccine RSVs trend by month and year. In nearly every year (8 out of 12), we saw a similar pattern with a significant rise in searches (ie, RSVs) leading up to July and August, followed by a drop in searches, suggesting that HPV vaccine interest was short-lived. This pattern of RSV peaks in July/August syncs with the annual school calendar and the back-to-school period. This is worth noting, as many states and school districts continue to weigh the benefits and costs of vaccine mandates, and whether the HPV vaccine will be required for school attendance [41]. Moreover, public health campaigns can partner with schools during this period to raise public awareness, strengthen parental knowledge, and offer HPV vaccination to all eligible students. This also has important implications, as public health authorities can use this period to promote public health campaigns through internet-based media. There was a considerable decrease in RSVs at the beginning of 2020, corresponding to the start of the COVID-19 pandemic, which could have diverted public interest away from the HPV vaccine onto COVID-19. Nevertheless, our findings showed a positive trend in the online interest of HPV vaccine health–seeking behavior from 2010 to 2021.

HPV vaccine searches differed by US states and demonstrated wide variations in year-over-year searches. All of the US states showed a positive trend in annual HPV vaccine searches from 2010 to 2021; however, some states such as Delaware (mean 23.1, SD 7.3), North Dakota (mean 22.8, SD 6.2), South Dakota (mean 22.4, SD 6.2), Vermont (mean 21.3, SD 6.2), and Wyoming (mean 17.4, SD 8.5) recorded lower RSVs. Although the underlying reasons for these differences are not clear, the changing trend in HPV vaccine searches indicates a positive impact on health-seeking behavior. In this regard, information technology interventions may consider targeting states with lower search volumes to raise awareness, or, alternatively, targeting states with higher search volumes to provide resources for action. Overall, targeted health education materials are needed to ensure that accurate, reliable, and updated information on the HPV vaccine is available online for parents, caregivers, adolescents, and young adults.

Our study identified variations in HPV vaccine search volume by time and geography. These findings could be used to inform targeted search engine advertisements that describe the benefits of the HPV vaccine and how it can prevent cancers, tailoring to different times, geographies, and topics. This approach builds upon prior work using Google Ads to deliver health education materials based on keyword searches [42,43] and geography [44]. For example, our findings demonstrate sharp increases in search volume after changes to HPV vaccine administration, such as making the vaccine available to boys or altering to a 2-dose series. When future changes or announcements arise, we may anticipate a large increase in online searches and create targeted and tailored messaging, utilizing Google Ads, to provide health education materials in the same space people are using to seek information. This approach can be tailored by geography (ie, targeting ads to searches from specific states) or even by topic (ie, tailoring ads to address vaccine safety, age eligibility, or misinformation, to name a few). Targeted online ads may also be utilized to link online searches to community resources or health care providers in local areas.

### Limitations

This study has several limitations. First, GT data are observational data; therefore, making causal inferences (eg, more HPV vaccine searching leads to greater HPV vaccine coverage) is not possible. However, we can use these infodemiology data, demonstrating variation in online searches by time and topic, to tailor health education and promotion materials related to HPV vaccination. These materials may be made available online and even targeted as search engine advertisements during periods of high search volume. Second, our study’s units of analysis were at the national and state levels. While these data provide overall indicators for online searches and vaccine coverage, they do not capture the relationship that may be present at other levels of analysis, such as at the community or county level. Third, we are unable to determine the true causes behind changes in search volume; that is, we do not know exactly what prompted increases or declines in searches, but we are able to surmise potential associations based on known federal guideline changes and other information. Fourth, our findings are biased in that they only represent individuals who have internet access and who use Google as their search engine. While most internet users use Google as their search engine (90%) [45], this does not represent the entire US population and may overrepresent certain types of individuals. Finally, variability in the data in specific years may have resulted in the statistical software incorrectly identifying joinpoints.

### Conclusions

This study supports the growing body of work examining online and other digital data, and their application to health care and public health research. Specific to the HPV vaccine, we examined GT data to document online search trends from 2010 to 2021. Our observational findings can be used to inform online intervention points such as event-based opportunities (ie, back-to-school night) and state-specific programs. Notably, we observed a marked decline in online searches during the start of the 2020 COVID-19 pandemic. Further investigation is needed to understand whether the significant factors and variations observed in our study hold to HPV vaccination trends outside of the United States.

## Abbreviations

ACIP: Advisory Committee on Immunization Practices
APC: annual percentage change
GT: Google Trends
HPV: human papillomavirus
MPC: monthly percentage change
RSV: relative search volume
