# Correction: Google Trends on Human Papillomavirus Vaccine Searches in the United States From 2010 to 2021: Infodemiology Study

**DOI:** 10.2196/42812

**Published:** 2022-10-04

**Authors:** Akshaya Srikanth Bhagavathula, Philip M Massey

**Affiliations:** 1 Center for Public Health and Technology Department of Health, Human Performance, and Recreation, College of Education and Health Professions University of Arkansas Fayetteville, AR United States

In “Google Trends on HPV vaccine searches in the U.S. from 2010 - 2021: An Infodemiology Study” (JMIR Public Health Surveill 2022;8(8):e37656) the authors noted one error.

In the originally published article, Figure 2 appeared incorrectly (as shown in Multimedia Appendix 1).

The correct Figure 2 is provided below.

The correction will appear in the online version of the paper on the JMIR Publications website on October 4, 2022, together with the publication of this correction notice. Because this was made after submission to PubMed, PubMed Central, and other full-text repositories, the corrected article has also been resubmitted to those repositories.

**Figure 2:**
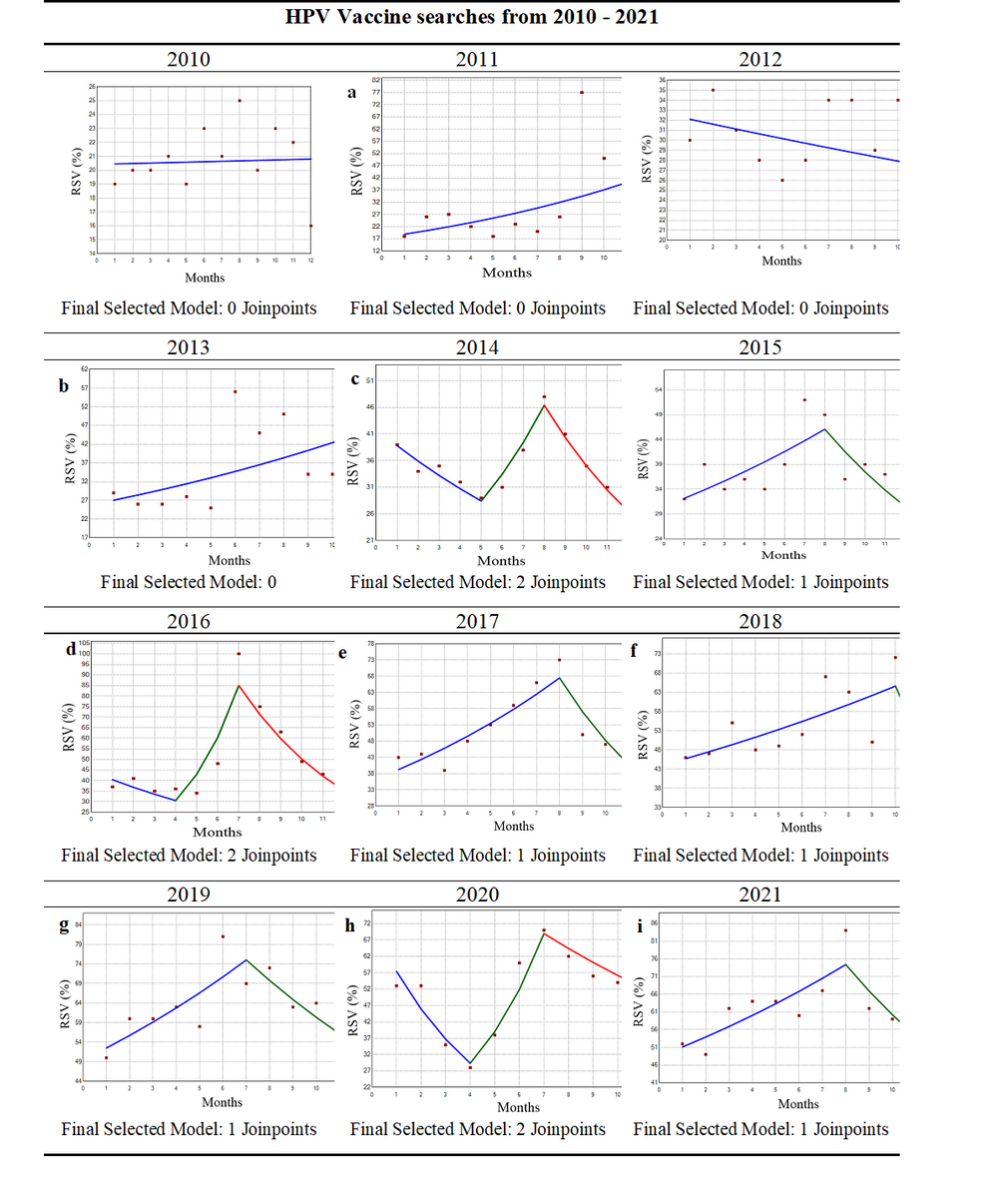
Joinpoint regression analysis indicating trends in HPV vaccine Relative Search Volume (RSVs) on Google Trends from 2010 – 2021 in the U.S.

## Appendix

Multimedia Appendix 1 Originally published Figure 2.

