# Supplementary figures and images for: Correction: Google Trends on Human Papillomavirus Vaccine Searches in the United States From 2010 to 2021: Infodemiology Study
Source: JMIR Public Health Surveill. 2022 Oct 4;8(10):e42812. doi: 10.2196/42812 (PMC9579932; doi:10.2196/42812)

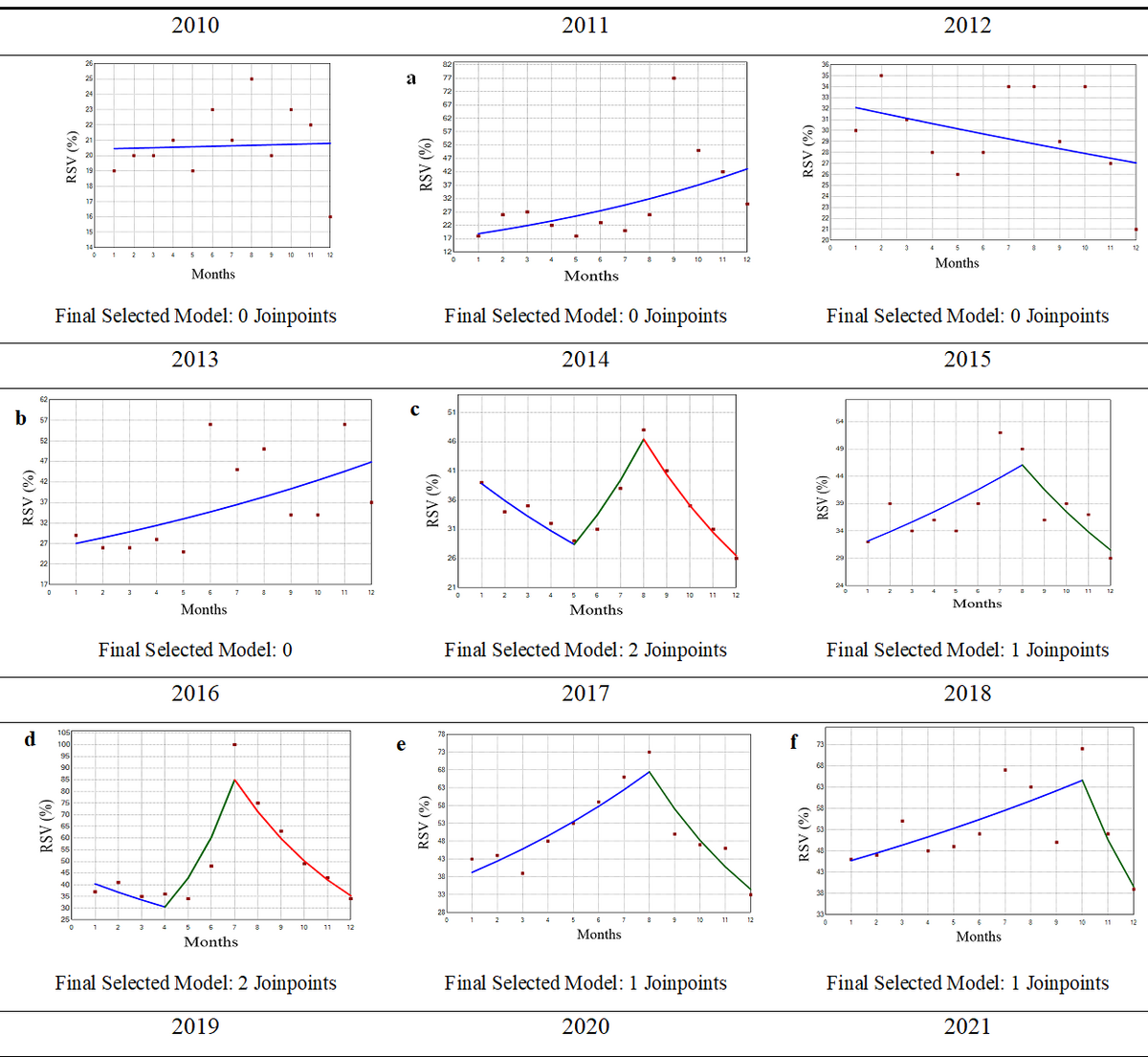

Supplement: Multimedia Appendix 1 [file publichealth_v8i10e42812_app1.png]
